# Whither dominance? An enduring evolutionary legacy of primate sociality

**DOI:** 10.1017/pen.2023.13

**Published:** 2024-01-08

**Authors:** Drew M. Altschul

**Affiliations:** 1 The University of Edinburgh, UK; 2 Scottish Primate Research Group, UK

**Keywords:** assertiveness, dominance, great apes, hierarchy, leadership

## Abstract

This article discusses dominance personality dimensions found in primates, particularly in the great apes, and how they compare to dominance in humans. Dominance traits are seen in virtually all primate species, and these dimensions reflect how adept an individual is at ascending within a social hierarchy. Among great apes, dominance is one of the most prominent personality factors but, in humans, dominance is usually modeled as a facet of extraversion. Social, cultural, and cognitive differences between humans and our closest ape relatives are explored, alongside humanity’s hierarchical and egalitarian heritage. The basic characteristics of dominance in humans and nonhuman great apes are then described, alongside the similarities and differences between great apes. African apes live in societies each with its own hierarchical organization. Humans were a possible exception for some of our history, but more recently, hierarchies have dominated. The general characteristics of high-dominance humans, particularly those living in industrialized nations, are described. Dominance itself can be subdivided into correlated subfactors: domineering, prestige, and leadership. Various explanations have been posed for why dominance has declined in prominence within human personality factor structures, and several possibilities are evaluated. The value of dominance in personality research is discussed: dominance has links to, for instance, age, sex, aggression, self-esteem, locus of control, stress, health, and multiple socioeconomic status indicators. The piece concludes with recommendations for researchers who wish to assess dominance in personality.

Primates tend to live in hierarchically organized social groups, and personality dimensions capturing dominance, assertiveness, and confidence are found in most primate species, particularly in the great apes (Freeman & Gosling, 2010). Much like humans, primates have a variety of major personality domains, such as extraversion, agreeableness, conscientiousness, emotionality, and dominance. Dominance generally refers to a personality trait of striving to be in high-status positions within social hierarchies, capturing behavior that enables an individual to both rise and maintain position in hierarchies (Mast & Hall, 2017). Dominance is distinct from social rank, an individual’s position within a hierarchy. As a personality trait, dominance is stable across time, but rank is circumstantial and malleable.

Dominance personality dimensions are found in humans as well: dominance, assertiveness, social potency, and self-confidence, to name a few. A comparable domain can be tapped by most broad personality inventories, but these constructs are generally less prominent in humans, e.g. dominance is not one of the Big 5, though it is an aspect of extraversion (DeYoung, Quilty & Peterson, 2007). This is not necessarily an impediment for measurement, and in contrast, the Abridged Big 5-Dimensional Circumplex (Hofstee, De Raad & Goldberg, 1992) presents dominance as a 2D rotation between agreeableness and extraversion, equivalent to the well-known Interpersonal Circumplex. See table/box 1 for key terms and definitions.

Ultimately, when one looks at human personality from the bottom up, one must go to some lengths to extract a dominance dimension. In nonhuman primates, the situation could hardly be more different. Among the great apes, for example, dominance is the first factor or component extracted by factor analysis or principal components analysis in all species (Eckardt et al., 2015; J. E. King & Figueredo, 1997; Weiss et al., 2015) with the exception of orangutans *(Pongo* spp.*)*, where extraversion comes first and dominance comes second (Weiss, King & Perkins, 2006). This piece asks whither dominance – what became of it? How is dominance in humans different, such that a once imposing, ubiquitous domain has been relegated to being a minor facet?

**Box 1.** Key dominance relevant constructs and their definitions.

Term	Definition
Social hierarchy	A system of linear variations in prestige, status, and authority among group members, also known as a “pecking order.” Describes who is influential and who submits to that influence.
Rank	Also known as “dominance rank.” An individual’s quantitative position in a social hierarchy. Circumstantially determined and subject to change in concert with the social conditions of the group.
Dominance	Short for “trait dominance.” A general personality trait, persistent and stable, that captures a tendency to prefer being in a high position in a social hierarchy. This trait applies to both human and nonhuman primates. Involves tendencies to be assertive, forceful, and self-assured, and as such, overlaps with trait assertiveness, confidence, social potency, boldness, etc.
Assertiveness	An adaptive style of communication in which individuals express their feelings and needs directly while maintaining respect for others. In some personality models, assertiveness is a facet of extraversion and has considerable conceptual and measurement overlap with dominance, social potency, etc.
Extraversion	A general personality trait characterized by personal orientation toward the outer world of living (and non-living) things. Dominance/assertiveness is one aspect and facet.
Social class	A collection of individuals with defined membership and privileges in the form of status, income, or property.
Social power	Or just “power.” An individual’s capacity to influence others, even when they resist. Derives from various sources including control over rewards, punishment, others’ attraction to powerful individuals, the powerholder’s access to superior information, etc. Resource-holding power is a subset. Often discussed in the context of motive traits, as a “need for power.”
Domineering	A trait indicating a desire to have high social control and coerce others into adhering to one’s will.
Prestige	A trait concerning the desire to be admired and respected primarily for one’s skills and knowledge.
Leadership	A trait capturing the desire to take initiative and responsibility in one’s group to direct it toward a common group goal.

## 1. The basics of rank-based societies

Dominance traits cannot be discussed without saying a bit more about the social systems in which they evolved. Rank-organized societies are found throughout the animal kingdom (Tibbetts, Pardo-Sanchez & Weise, 2022), along with personality traits relating to social potency. I will focus here and throughout the rest of the piece on primates, and on our closest evolutionary relatives, in particular, great apes.

Hierarchies are typically perpetual: the structure exists beyond the lifespan of any member, and for this to work, hierarchies must be plastic. New groups and hierarchies can still spring up while others disperse, however. For these circumstances, there are mechanisms whereby a hierarchy can be established (Tibbetts et al., 2022). Existing hierarchies must be entered by an individual at some point in their life, usually on the cusp of adulthood. The most common way for individuals to establish themselves in hierarchies and then maintain or even rise in rank is through competitive dyadic interactions.

Competitive dyadic interactions are the computational basis for dominance hierarchies (Bernstein & Blue, 2019). Even solitary yet social species like orangutans exhibit asymmetrical dyadic relationships (Knott et al., 2008), in which one individual somehow demonstrates their power over another individual. When a competitive bout occurs, the individual with greater resource-holding power (RHP), akin to fighting prowess, and higher motivation is more likely to win the bout (Qu, Ligneul, Van der Henst & Dreher, 2017). If the winner is lower ranked, it will gain status and may move up in rank; if the winner is higher ranked, its status will be further cemented. Hierarchies need to be malleable, but is generally beneficial for them to be stable across time; stable hierarchies help a group stay together and succeed (Tibbetts et al., 2022). Stability is associated with less conflict (Forkman & Haskell, 2004), better individual health (Sapolsky, 2004), and impaired group-level functioning (Maldonado-Chaparro, Alarcón-Nieto, Klarevas-Irby & Farine, 2018).

Depending on the species and particular society, individuals may leave their natal group and disperse to a new group, or they may stay in their natal group. For instance, in *Pan*, both chimpanzee and bonobo females disperse (Koenig & Borries, 2012). This then impacts how individuals navigate dominance hierarchies, e.g. chimpanzee females enter their own distinct hierarchy at a low rank, and queue to achieve higher status as higher ranking, usually older, females die (Foerster et al., 2016). Chimpanzee males enter their hierarchy around age 12, typically rise in rank before falling as they age, and lose the RHP necessary to win dyadic bouts (Weiss et al., 2023). Bonobo males follow a similar pattern, but their status is deeply intertwined with that of their mother (Furuichi, 1997; Surbeck, Mundry & Hohmann, 2010). Therefore, the bonobo male’s status depends on how effectively their mother integrated into the postnatal group after dispersing.

Rank societies, perhaps particularly in primates, are notable for their inclusion of coalitions. Coalitionary behavior is found across the primate order and usually refers to when two or more act together against a third party in a competitive context (Harcourt & de Waal, 1992). Alliances are essentially long-term coalition-based relationships, usually between two individuals, though human alliances can of course be much larger (Bissonnette et al., 2015). Coalitions can also occur opportunistically, seemingly in the moment. Coalitions play an important role in both intragroup and intergroup dynamics. Group-wide coalitions might come into play when there is conflict between groups, such as when male chimpanzees patrol the perimeter of their territory and sometimes fight males from other groups (Watts & Mitani, 2001). Within a group, a coalition of lower-ranking males might form in order to oust an alpha, or individuals might ally themselves with the alpha in order to benefit from the alpha’s status (Feldblum, Krupenye, Bray, Pusey & Gilby, 2021). Females form coalitions as well, though more frequently among bonobos, as female chimpanzees are less gregarious by comparison (Mitani, 2009). Despite not being directly related, female bonobos readily form coalitions with other females, usually to attack males that have been aggressive to someone in the coalition (Tokuyama & Furuichi, 2016), which may theoretically prevent the rise of despotic male bonobos (Ronay, Maddux & von Hippel, 2020). Male bonobos form coalitions in the context of within-group conflict as well, though they form most coalitions with females (Surbeck et al., 2017).

Coalitional social bonds are related to hierarchical rank as well. Male chimpanzees in coalition leverage their relationships in order to maintain or rise in rank (Bray, Feldblum & Gilby, 2021). It is male bonobos’ coalitions with their mothers that influence male rank in the group, despite bonobos having male-philopatric societies (Surbeck et al., 2010). For a more complete treatment of coalitions and alliances, see Bissonnette et al. (2015).

Since personality is stable and rank changes over time, it can be difficult to relate trait dominance to transient rank. Moreover, both are assessed through entirely different techniques. A recent study that has grappled with this using long-term data from male chimpanzees found that trait dominance was significantly related to rank, throughout the individuals’ lifetimes, though this relationship was particularly strong during certain stages of the chimpanzees’ lives, specifically, early to middle adulthood (Weiss et al., 2023).

## 2. The differing sociocognitive landscapes of primates

The African apes – chimpanzees *(Pan troglodytes)*, bonobos *(Pan paniscus)*, and gorillas *(Gorilla* spp.) – all live in rank-based social groups, but the ways in which each species, and even each sex within species, operate their dominance hierarchies differ considerably. Chimpanzee males compete with each other for status and eventually lose rank status with age, whereas chimpanzee females, as noted earlier, enter low and rise in a queue (Foerster et al., 2016). Bonobo hierarchies are less distinct: females wield greater power in bonobo society, so much so that bonobo hierarchies appear to be sex-independent (Surbeck & Hohmann, 2013). Bonobos are nepotistic and males are philopatric; as noted, a male’s status depends in significant part on the status of their female relatives (Surbeck et al., 2010). Bonobo males and females are best described as “co-dominant.” Gorilla groups can be multi-male (bachelor groups), multi-female single-male (silverback male dominating a harem), and multi-male multi-female. Within harems, some gorilla females seem to develop hierarchies (Robbins, Gerald-Steklis, Robbins & Steklis, 2005) whereas others may not (Stokes, 2004). Within multi-male multi-female groups, gorilla males were organized in a stable linear hierarchy, whereas in a bachelor group, there was no clear hierarchy, only a clear alpha male (Robbins, 1996).

Human social systems are the most studied, but probably least understood, particularly in terms of their evolution. The pre-eminent theory of human social evolution argues that at some point between when we diverged from chimpanzees and bonobos to the end of prehistory, homininians (bipedal apes) adopted egalitarianism as the primary form of social organization (Gintis, van Schaik & Boehm, 2015). Egalitarianism still exists today in some cultural groups outside of the industrialized world, like the Hadza people of Tanzania. From these groups, anthropologists have established that egalitarianism requires a reverse dominance hierarchy (Erdal, Whiten, Boehm & Knauft, 1994), a culture in which economic equality is encouraged through resource sharing – the so-called “gift economy.”

In nonhumans, aggression, strength, and proxies for physical power (e.g. physical height) are used in fights to establish themselves in a rank hierarchy, though even among nonhumans, fights are often ritualized to an extent, enabling individuals to avoid the worst physical harm. With the advent of egalitarianism, social norms developed that enforced reverse dominance hierarchies, wherein no individual could gain status over another individual. Males and females typically inhabit separate hierarchies, so this does not imply that males, females, or children are inherently equal (Flanagan, 1989). Hoarding, displays of authority, and aggression are discouraged among egalitarians. Unacceptable behavior is punished with shunning, exile, and lethal violence is reserved for putting down upstarts who might challenge egalitarian norms (Wrangham, 2019). Ironically, a new role might have been created for “cooperative” dominant individuals (Chen, Zhang, Laustsen & Cheng, 2021), such as police and executioners. For extended treatment on this topic, see Christopher Boehm’s “Hierarchy in the Forest” (Boehm, 1999).

Nevertheless, modern humans display a strong proclivity for hierarchical living. Virtually all post-industrial societies are organized hierarchically, on the basis of various indicators of social status, but especially income and wealth. While western, educated, industrialized, rich, and democratic humans may not represent our ancestral condition, living forager cultures are frequently used as proxies for early humans. In an ethnographic study of 60 societies, respecting superiors, that is, “being deferential, respectful, loyal, or obedient to those above you in a hierarchy” was viewed as a virtue in 133 attestations, and never viewed negatively (Curry, Mullins & Whitehouse, 2019). Moreover, the same study found that traditional societies valued “hawkishness,” that is, one’s ability to contest with others using strength, boldness, skill, and intelligence; hawkishness, too, closely aligns with dominance traits.

Though many modern societies have grown increasingly unequal (Ronay et al., 2020), which leads to more opportunities for hierarchical comparisons to others, this development does not necessarily imply that dominance traits have become more influential again. Aggression and warfare have steadily decreased over history (Pinker, 2011), violent acts of dominance are policed, and perpetrators are punished. The punishment is not the same as among egalitarians, but spending time in prison is an impactful sentence. Modern society leaves little room for ape-like manifestations of dominance.

## 3. What are the dominance traits in nonhuman primates?

Among the African apes, the dominance dimension shares many items in common, for example, “independent,” “persistent,” and again “dominant,” but not always “timid” or “submissive.” Though it is less prominent, dominance is found in orangutans, white-faced (*Cebus* spp.) and brown capuchins (*Sapajus apella*) (Robinson et al., 2016), Tonkean, Japanese, black, and Barbary macaques (*Macaca* spp.) (Adams et al., 2015; Baker, Lea & Melfi, 2015), squirrel monkeys (*Saimiri* spp.) (Baker et al., 2015), marmosets (*Callithrix jacchus*) (Koski et al., 2017), and Hanuman langurs (*Semnopithicus* spp.) (Konečná et al., 2008). In rhesus macaques, there are two dimensions capturing social potency: a more traditional dominance dimension, and one labeled confidence, which describes individual monkeys who are neither “timid,” “vulnerable,” “submissive,” nor “dependent/follower(s)” (Adams et al., 2015; Weiss, Adams, Widdig & Gerald, 2011), thus capturing aspects of emotional stability.

The literature on primate personality is considerably smaller than the literature on human personality, so, to date, studies have not deconstructed the single dominance trait of apes into lower-order facets. Nevertheless, various more advanced, human-like manifestations of dominance have been observed in great apes. Higher-ranking individuals often lead group movements (A. J. King, Johnson & Van Vugt, 2009). In bonobos, higher-ranking males and older, presumably more knowledgeable females were more likely to initiate group movements in a particular direction. (Tokuyama & Furuichi, 2017). Chimpanzee males follow a similar pattern, with the highest-ranking males more likely to lead group movements and patrols (Goodall, 1986). However, these leaders’ goals are usually highly self-serving, e.g. an alpha male chimpanzee may lead his group to a concentrated foraging site that he could monopolize in order to maximize his food consumption, whereas the group as a whole would benefit more from visiting a distributed foraging site (Ronay et al., 2020).

Prestige as freely conferred deference is not a concept yet identified in apes, however, prestige bias is present: during social learning experiments, chimpanzees prefer to copy an individual who has a prior track record of success, is older, and higher ranking (Horner, Proctor, Bonnie, Whiten & Waal, 2010). Moreover, when a new task is introduced and certain individuals display greater skill with the task, those individuals also tend to attract more attention and grooming from conspecifics (Lee & Yamamoto, 2023).

## 4. What are the dominance traits in humans?

On its face, dominance in humans is captured by adjectives such as “assertive,” “forceful,” “outspoken,” and of course “dominant.” It is often represented by items indicating that an individual wishes to have control and power over others. It is also represented by items indicating a desire for leadership, influence, and attention. Many classical inventories have assumed that dominance and leadership traits are distinct but overlap in content, and recent empirical work (Altschul & Moore, 2023a) suggest that many scales labeled “dominance,” “leadership,” or “assertiveness” all measure the same thing. The opposite of dominance is “submissiveness,” which represents the opposite pole on the Interpersonal Circumplex, for instance. Submissiveness is negatively correlated with dominance to the same approximate degree as other positively related constructs, like social potency and leadership (Altschul & Moore, 2023a).

In addition to being found across species, dominance, via NEO-PI-R assertiveness, is found across human cultures, too. Assertiveness is reliable in at least 24 cultures (De Fruyt, De Bolle, McCrae, Terracciano & Costa, 2009), although across cultures in both adolescents and adults, assertiveness loaded comparably and significantly (and negatively) on agreeableness, as well as extraversion (De Fruyt et al., 2009; McCrae & Terracciano, 2005), which aligns with the Abridged Big 5-Dimensional Circumplex perspective (Hofstee et al., 1992).

Human dominance can be broken down into smaller facets. Despite the fact that many inventories are not able to reliably capture lower-level subfactors of dominance, more recent, concerted efforts have been more successful. Cheng and colleagues demonstrated the distinct role of two strategies, dominance and prestige, in the attainment of high rank (Cheng, Tracy & Henrich, 2010; Cheng, Tracy, Foulsham, Kingstone & Henrich 2013; Redhead, Cheng, Driver, Foulsham & O’Gorman, 2019). For clarity, this piece will refer to what Cheng and colleagues term dominance as “domineering” in order to distinguish it from the broader dominance trait used throughout the personality literature. Suessenbach, Loughnan, Schönbrodt, and Moore et al. (2019) linked trait dominance to the power motive and established that all-encompassing dominance or power motivation can be broken down into domineering, prestige, and leadership (DoPL) traits. Domineering is oriented around controlling others and getting them to do what you want through force and coercion. Prestige involves accomplishment and achievement, and status gained through prestige is freely given, not taken. Prestige is less closely related to the other traits and is arguably related to the achievement motive as well (Schönbrodt & Gerstenberg, 2012). Nevertheless, an individual who tends to be prestige-motivated also tends to be domineering. Leadership captures how much an individual wants to be in charge and how often they lead, as well as how good they think they are at leadership. Power gained through this avenue is granted, by the group as a necessity, as well as claimed by the individual in order to achieve a group goal.

Domineering and leadership may seem like “bad” and “good” types of dominance, respectively, but this is not the case. Leaders tend to be physically stronger (though not aggressive), perceive themselves to be higher in status and have more social capital, and be ruthlessly self-advancing (Altschul & Moore, 2023a) – this suggests a coercive style of leadership (von Rueden, 2020). Rather than thinking of these traits as good or bad, one ought to think about the context in which each trait would be most useful. Being domineering is useful in one-on-one situations, when an individual can use intimidation and force to take what one likes, or even get another individual to follow orders. This might be termed “dyadic dominance,” which is in keeping with the dyadic interaction foundations of animal social hierarchies (Drews, 1993). Leadership is most useful in a group. When an individual is outnumbered and force is not practical, charisma and coercion are much more effective at getting others to do what one wishes. This might be termed “group dominance.”

## 5. What are the characteristics of dominant humans?

Dominance, assertiveness, leadership, and other social potency constructs in humans all typically measure a general disposition to be exactly what these labels suggest, with considerable overlap between the constructs (Altschul & Moore, 2023a), thus raising the possibility of a “jangle” fallacy. For instance, with an adjectival approach, a dominance construct might be defined by “dominant,” “assertive,” “outspoken,” and “forceful,” whereas a leadership construct defined by statements might include “I want to be in charge,” “I try to lead others,” and “I can talk others into doing things.” In this way, the traits have decent face validity, even though many measures lack specificity for, say, leadership abilities. But does a dominant human act like a dominant chimpanzee? Or bonobo, or gorilla?

Cross-species evidence in this area is lacking, particularly on the DoPL lower-order factors. One study suggests that “fearless dominance,” a broad trait derived from the psychopathic personality inventory, captures the character of general primate dominance in humans (Weiss, 2022). Extraversion’s facet/aspect of assertiveness is another strong contender for capturing a general dominance factor (Altschul & Moore, 2023a).

The wider network of associations with dominance is beginning to be revealed in humans. Connections are apparent between dominance, hubristic pride, narcissism, and Machiavellianism (Altschul & Moore, 2023a; Cheng et al., 2010), as well as with aggression and anger (Altschul & Moore, 2023b). Dominant individuals are immodest, exhibitionist, self-deceiving, and less concerned about harming others or fairness, but more concerned about favoring their in-group (Suessenbach et al., 2019).

On the positive side, higher dominance is also associated with more internalized locus of control, higher self-esteem (Altschul & Moore, 2023a), and higher affect (Altschul & Moore, 2023b). Assertive individuals are charismatic (House & Howell, 1992), speak more charismatically (Michalsky, Niebuhr & Penke, 2020), and domineering individuals utilize distinct patterns of nonverbal communication (Witkower, Tracy, Cheng & Henrich, 2020). Dominant leaders appear to instill more cooperation in their followers (Chen et al., 2021). Dominant individuals also showed faster reaction times, but poorer reasoning in one study (Graham & Lachman, 2014), but better executive functioning in another (Altschul & Moore, 2023b).

Dominance is also associated with physiological traits. More dominant individuals have wider faces (Lefevre, Etchells, Howell, Clark & Penton-Voak, 2014), a result consistent with, but stronger in primates (Altschul, Robinson, Coleman, Capitanio & Wilson, 2019; Martin, Staes, Weiss, Stevens & Jaeggi, 2019; Wilson et al., 2020). Assertiveness is associated with higher body mass index (Sutin, Ferrucci, Zonderman & Terracciano, 2011) and leptin, the major hormone controlling hunger (Sutin et al., 2013), as well as higher basal metabolic rate (Arumäe, Mõttus & Vainik, 2022).

Altogether, this collection of associations converges well along key lines. Dominants are physically larger and stronger, more socially powerful and influential – both coercively and charismatically. More dominant individuals have higher RHP (Altschul & Moore, 2023a). Dominance in humans thus seems highly reminiscent of dominance in nonhuman primates.

## 6. How dominance traits are the same in humans and nonhuman primates

Above, I identified a host of dominance traits in nonhuman primate species. But just because traits have been labeled the same way does not make them the same thing – the so-called “jingle fallacy” – particularly when one is crossing species boundaries (Zuckerman, 1992). Fortunately, there are many similarities in dominance between humans and nonhumans; I will focus on comparisons with our closest ape relatives.

Dominance is often associated with masculinity. Males are widely known to be more aggressive, particularly with direct, physical aggression (Archer, 2004); this difference is reflected further downstream with the advent of “male dominated” violent crime (Steffensmeier, 1980). In trait terms, dominance is higher in men than women (Del Giudice, Booth & Irwing, 2012). The same holds true for chimpanzees and orangutans: males rate higher on dominance than females (Weiss & King, 2015). It is notable, however, that in bonobos, males rate lower on assertiveness than females (Staes, Eens, Weiss & Stevens, 2017). This is in keeping with bonobo socioecology, where females occupy higher ranks than males (Furuichi, 2011), and goes to show that structural differences in society can have major impacts on social behavior and associated norms.

Age is a highly relevant factor for dominance as well. As noted above, male chimpanzees compete for high rank, whereas female chimpanzees enter their hierarchy low and work their way up (Foerster et al., 2016). In terms of traits, both male and female chimpanzees exhibit positive relationships between dominance and age, and the same is true for orangutans (Weiss & King, 2015). However, bonobos buck the trend once again, for there appears to be no association between age and assertiveness in bonobos (Staes et al., 2017), although higher assertiveness bonobos do appear to be higher ranked (Franz, 1999; Furuichi, Thompson & Fruth, 2008). Age is not as relevant factor for human dominance traits. Considerable evidence suggests that dominance increases with age (Roberts, Walton & Viechtbauer, 2006), while other evidence suggests that it is flat across adulthood (Soto, John, Gosling & Potter, 2011).

Dominance is also heritable. Evidence from human twin studies (Figueredo, Vasquez, Brumbach & Schneider, 2004; Jang, McCrae, Angleitner, Riemann & Livesley, 1998), and chimpanzee (Weiss, King & Figueredo, 2000), bonobo (Staes et al., 2016), and orangutan (Adams et al., 2012) pedigree analyzes suggests at least moderate heritability (*h* = 0.22 – 0.63). Although these metrics should assess genetic contribution, since dominance and hierarchy are strongly driven by complex familial social relationships, this should not be taken as the final word on the nature of heritability.

## 7. Where did dominance “go” and why?

Although this, like other adaptive, teleonomic questions, is essentially impossible to answer, it is important to spell out why. Behavior does not fossilize, apart from indirect, fragmentary evidence like marks on bone or footprints. We can know nothing certain about the behavior of our recent and more distant hominoid ancestors, thus we cannot trace the trajectory of human behavior through recent human biological, as well as cultural, evolution. However, relevant evidence exists that can nevertheless inform us on this topic.

### 7.1 The traditional account: egalitarianism subverted dominance

The traditional take on hunter-gatherer egalitarianism suggests that dominance became less relevant to humans because for hundreds of thousands of years we lived in reverse dominance hierarchies. No individual could attain a rank above another (Boehm, 1999). Our psychology and cultural niches have been shaped by egalitarian conditions.

This perspective has several problems, however. First among them is that the egalitarian hypothesis has been increasingly criticized (Flannery & Marcus, 2012; Singh & Glowacki, 2022). Singh and Glowacki (2022) argue that the recent prehistory of hominoids was filled with diverse social structures. Habitat variability, such as temporal shifts in climate and environment, was a circumstance to be adapted to, not borne through under the same, rigid social structure. Ethnographic evidence from modern and historic cultures demonstrates that spatiotemporal resource distribution is linked to greater and lesser inequality (Ronay et al., 2020; Smith & Codding, 2021). When resources are concentrated they can be monopolized, and in humans, monopolizability seems to lead to a wholesale shift in the balance of a social system, from egalitarian to hierarchical and despotic. Further, in the archaeological record, material signs of hierarchy, such as richly adorned, lavishly buried human remains, are found in some of the earliest known remnants left by anatomically modern humans (Flannery & Marcus, 2012). Throughout human history, a wide variety of more and less unequal societies have existed and are possible.

There are psychological objections to this account as well. Even if egalitarianism dominated human social structure for hundreds of thousands of years, hierarchy was not out of mind. Egalitarians participated and still participate in supernatural hierarchies consisting of divinities at the top, ancestor spirits beneath them, and living humans at the bottom (Flannery & Marcus, 2012). This belief was continuous with later chiefdoms and kingdoms, where the leaders were often speaking on behalf of divinity, or related to divinity through descent. These individuals drew their power from their relationships with the “true alphas” – the gods (Graeber & Sahlins, 2017). Moreover, these examples do not speak to within-family hierarchies – grandparents above adults, adults above children, men above women – and as was noted earlier, a prime virtue among traditional societies is to respect one’s elders (Curry et al., 2019).

### 7.2 Dispersing dominance across factors

Weiss (2022) argues that the great ape dominance factors dissipated during human evolution and can now be found spread across different facets of different domains of the Big 5. Fearless dominance, which is a broad construct drawing on, in particular, facets of extraversion, neuroticism, and openness, is, for this reason, a good fit as a human analog of great ape dominance. Fearless dominance converges with a general factor of dominance or power-seeking, as well as any other general construct of dominance or assertiveness (Altschul & Moore, 2023a). Fearless dominance itself is strongly associated with low behavioral inhibition, high sensation and fun-seeking, low anxiety and internalizing, and narcissistic personality disorder (NPD) diagnosis.

Why might dominance be dispersed across facets? The lexical method for deriving personality, i.e. using language and questions to tap into traits, is impacted by norms and morals inherent to the culture of the individual, and the language(s) of that culture (Saucier, 2018). Our norms against dominance and the lack of objective language may be getting in the way of our ability to capture this trait with the lexical method. For instance, I would say that when chimpanzee makes an act of their physical prowess that individual is “displaying,” but if a human makes a similar act, I might say that the individual is “making a scene,” which coveys a clear judgmental stance on the normative acceptability of the act. One might also describe a human acting in such a manner as having a “fit” or “tantrum,” both of which evoke immaturity and mental instability, which are also normatively undesirable. The core of what it could mean to be dominant might thus be broken up and attached to different normative, moral distinctions that align with different facets under the Big 5.

### 7.3 The dark triad

Much of modern personality science examines morality through the lens of dark personality traits. As noted earlier, the wider nomological net of dominance includes narcissism and Machiavellianism (Altschul & Moore, 2023a), and fearless dominance is drawn from the psychopathic personality inventory (Lilienfeld, Gershon, Duke, Marino & de Waal, 1999). However, fearless dominance may actually capture the aspects of the psychopathic personality inventory that are not due to clinical psychopathy, which is captured by antisocial impulsivity (Miller & Lynam, 2012). Machiavellianism appears to have a particularly high association with the domineering facet, a smaller association with prestige, and no notable relationship with leadership (Schattke & Marion-Jetten, 2021; Semenyna & Honey, 2015).

Narcissism stands out. NPD is described as “pattern of grandiosity, need for admiration, and lack of empathy” (American Psychiatric Association, 2013); its correlation with general dominance is high (*ρ* = 0.5; Altschul & Moore, 2023a). Narcissism can be broken down into three facets: leadership/authority, grandiose exhibitionism, and entitlement/exploitativeness. Fearless dominance is highly correlated with leadership/authority and grandiose exhibitionism, but not entitlement/exploitativeness (McDonald, Donnellan & Navarrete, 2012). Furthermore, narcissists’ popularity starts strong and grows, but declines after some time (Leckelt, Küfner, Nestler & Back, 2015), resembling the relationship between dominance and rank in male chimpanzees (Weiss et al., 2023). The dominants of the past may have found a place in society as the less entitled narcissists of today.

### 7.4 The role of language

It might be more beneficial to look not at what our ancestors lost – rigid hierarchy – but at what they gained – language. Language bears directly on aspects of extraversion (Goldberg, 1992; Hofstee et al., 1992; Trapnell & Wiggins, 1990) that dominance has less to do with: “talkative,” “gregarious,” “sociable,” “verbal,” “wordy,” “communicative,” and (not) “quiet.” While it is difficult to scientifically say whether we are less hierarchical than our ancestors, we are certainly less aggressive (Wrangham, 2019), and lowered aggression plus faculty with language could have led to fighting, posing, and jockeying being replaced with all manner of conversation.

Moreover, with the advent of language came a move away from grooming. Being able to have more conversational partners meant that groups could become larger (Dunbar, 2017). Larger groups and being able to have more interaction partners at one time would have alleviated pressure on the individual because there would have been more real available interaction time to go around. Under these conditions, dominance relationships would become complex and possibly multidimensional. Individuals would likely become more specialized; some might become talented at skill-based activities and gain prestige, while others might excel at group organization, negotiation, and leadership. Rank relationships might become so complex that most individuals would be unable to keep track of the entire hierarchy or hierarchies.

Crucially, language moved conspecific interaction *away from the dyad.* An individual can only groom one other individual, but they can speak with two, three, four, or more individuals (Dunbar, 2017). The majority of interactions would be with groups. Nonhuman primate dominance is largely oriented around behaviors that are useful in competitive dyadic interactions, so these behaviors may have become less adaptive. Speaking involves less physical contact with one’s conspecifics than grooming. Being at a distance and less physically involved with members of one’s community might accompany a reduction in the inherent drive to display physically. Rather, displays of power would come to rely on language-based performance, which brings together physicality (domineering), skill, and respect (prestige), as well as charisma and persuasion (leadership).

It is possible, and likely, that more than one explanation for “whither dominance” is required. None of the above mechanisms are mutually exclusive, and none are definitive. Thus, the historical impact of egalitarianism and the development of language may have dispersed dominance across various facets, though clear dominant types, like narcissists, still exist and are easily visible in today’s societies.

## 8. Why study dominance?

One can argue about whether or not dominance has declined and how to even measure personality change over evolutionary time; regardless, dominance remains, and it remains relevant, perhaps more so today in what appears to be a burgeoning era of authoritarianism (Chen et al., 2021). Dominance exists in humans, and as presented in this review and elsewhere (Altschul & Moore, 2023a, 2023b), it is valid, reliable, and has unique associations with meaningful criterion variables.

Dominance is distinctive. A layperson can identify how dominant or submissive a peer is in a particular context; dominance has good face validity. Though there is overlap with extraversion and openness, dominance distinguishes itself through distinct associations with constructs like anger (Altschul & Moore, 2023b). Moreover, dominance may be more strongly related to constructs like self-esteem, locus of control, and achievement than broader extraversion (Altschul & Moore, 2023a).

As stated earlier, dominance is not rank. Rank is not class, either, though social classes (such as the British class system or Indian caste system) can incorporate rank (Pandit, Pradhan & van Schaik, 2020). If rank exists in humans, it is subtle and probably multifaceted. Distinct hierarchies may exist for the prestigious, domineering, and leaders, though since someone who is likely to be high in leadership is also likely to be high in prestige, that individual is likely to do well in multiple types of hierarchy (Altschul & Moore, 2023a; Suessenbach et al., 2019). In some social groups, certain traits may not be as influential – for example, there is probably little scope to get ahead by being domineering if you are an ascetic living in a monastic community. Nevertheless, nearly all humans live in hierarchically organized societies, and dominance is extraordinarily relevant to life in such societies. Humans may or may not have ranks the way nonhumans do, but we make frequent implicit status assessments from a young age (Heck, Shutts & Kinzler, 2022). When a human behaves dominantly, dyadically, or in a group, that individually is leveraging existing sociocultural structures and neural representations (Chiao, 2010; Cloutier, Cardenas-Iniguez, Gyurovski, Barakzai & Li, 2016; Ligneul, Obeso, Ruff & Dreher, 2016; Qu et al., 2017). As such, dominance is associated with key human socioeconomic status (SES) indicators, such as education and high income (Gensowski, Gørtz & Schurer, 2021). Moreover, the entire field of leadership studies is arguably wrapped up in dominance, so strong is the relationship among the constructs (Altschul & Moore, 2023a; Suessenbach et al., 2019).

In the nonhuman primate literature, dominance and rank have been studied extensively from a stress and health perspective (Sapolsky, 2005). Subordinate primates in particular appear to have higher cortisol when exposed to more stressors, and when they have less social support (Abbott et al., 2003). Personality’s relationship with health has been extensively studied in humans, although the best-known associations come from, for instance, neuroticism (Strickhouser, Zell & Krizan, 2017). However, dominance is, again, not often studied in this context apart from extraversion, and some studies suggest that submissiveness is associated with less risk of cardiovascular disease (Newton, 2009; Whiteman, Deary, Lee & Fowkes, 1997) and less stress (Altschul, 2018).

## 9. Recommendations and concluding thoughts

Dominance is not difficult to measure: a psychometric instrument using Likert scales that inquires about an individual’s dominance, assertiveness, outspokenness, timidity, and desire to lead will work. Many purported measures of dominance, assertiveness, etc suffice – see Altschul and Moore (2023a) for a comparison of many common measures. If you have access to NEO, Big 5, or HEXACO data, the facet or aspect structure of all these models (and others besides) will measure something akin to dominance. Standard recommendations regarding personality assessment apply, e.g. more items yield better measurement than fewer items. If you wish to assess a broader construct, i.e. fearless dominance, NEO, HEXACO, IPIP, and others inventories can also be specially scored up to construct this domain (Witt, Donnellan & Blonigen, 2009).

For the most comprehensive measure of dominance and its facets, the DoPL framework and questionnaires appear to be the best currently available (Suessenbach et al., 2019). These inventories measure general dominance as well as three subfactors: domineering, leadership, and prestige. The scales were developed in order to capture affective, behavioral, cognitive, and desire aspects, and among many recently developed instruments, these measures had the best psychometric properties; DoPL was the only dominance inventory readily useable in confirmatory factor analysis and structural equation modeling (Altschul & Moore, 2023a).

Together, broad fearless dominance and narrower assertiveness aspects and facets raise a question of what it means for the “importance” of a construct if it is easily identifiable, measurable, and found across multiple levels, but is not obviously present at the level revealed by the most convenient statistical model. On the other hand, more recent developments in the “nuance” oriented approach (Mõttus, Kandler, Bleidorn, Riemann & McCrae, 2017) and causal modeling (Deffner, Rohrer & McElreath, 2022) suggest that researchers ought to focus on the variables that are pertinent to their research question and think carefully about what covariates to include.

In conclusion, dominance is a widespread, meaningful personality construct. Even egalitarians possess differing amounts of a tendency to seek power, over others and themselves. All primates are, ultimately, hierarchical beings and dominance traits only make sense in the context of hierarchies and the individual’s pursuit of power and status. To understand our existence in the hierarchies we navigate, it is necessary to understand human, and nonhuman, primates’ individual psychological differences in dominance and submissiveness.
